# Pain-Relieving Effects of mTOR Inhibitor in the Anterior Cingulate Cortex of Neuropathic Rats

**DOI:** 10.1007/s12035-018-1245-z

**Published:** 2018-07-22

**Authors:** Sun Woo Um, Min Jee Kim, Joong Woo Leem, Sun Joon Bai, Bae Hwan Lee

**Affiliations:** 1 Department of Physiology, Yonsei University College of Medicine, 50-1 Yonsei-ro, Seodaemun-gu, Seoul, 03722 Republic of Korea; 2 Brain Korea 21 PLUS Project for Medical Science, Yonsei University College of Medicine, Seoul, Republic of Korea; 3 Department of Anesthesiology and Pain Medicine, Yonsei University College of Medicine, Seoul, Republic of Korea

**Keywords:** Neuropathic pain, Anterior cingulate cortex, mTOR, Rapamycin, Synaptic plasticity

## Abstract

The anterior cingulate cortex (ACC) is a well-known brain area that is associated with pain perception. Previous studies reported that the ACC has a specific role in the emotional processing of pain. Chronic pain is characterized by long-term potentiation that is induced in pain pathways and contributes to hyperalgesia caused by peripheral nerve injury. The mammalian target of rapamycin (mTOR) signaling, which is involved in synaptic protein synthesis, could be a key factor controlling long-term potentiation in neuropathic pain conditions. Until now, there have been no reports that studied the role of mTOR signaling in the ACC involved in neuropathic pain. Therefore, this study was conducted to determine the relationship of mTOR signaling in the ACC and neuropathic pain. Male Sprague-Dawley rats were subjected to cannula implantation and nerve injury under pentobarbital anesthesia. Microinjection with rapamycin into the ACC was conducted under isoflurane anesthesia on postoperative day (POD) 7. A behavioral test was performed to evaluate mechanical allodynia, and optical imaging was conducted to observe the neuronal responses of the ACC to peripheral stimulation. Inhibition of mTOR by rapamycin reduced mechanical allodynia, down-regulated mTOR signaling in the ACC, and diminished the expressions of synaptic proteins which are involved in excitatory signaling, thereby reducing neuropathic pain-induced synaptic plasticity. These results suggest that inhibiting mTOR activity by rapamycin in the ACC could serve as a new strategy for treating or managing neuropathic pain before it develops into chronic pain.

## Introduction

Neuropathic pain is induced by various causes, including tissue or nerve damage, and is characterized by symptoms of spontaneous pain, allodynia, and hyperalgesia [1]. Currently, many therapies such as antidepressants and antiepileptics are used to treat neuropathic pain, but these medications have side effects and are not always effective [2–4]. Thus, the management of neuropathic pain remains difficult. In the brain, cortical areas that process information related to pain are known as the “pain matrix,” which includes the anterior cingulate cortex (ACC), insular cortex (IC), thalamus, somatosensory cortex, and prefrontal cortex [1, 4, 5]. Among these regions, the ACC is the main area associated with pain perception [6], and accumulating evidence implicates the involvement of the ACC in the emotion processing of pain [7, 8].

Long-term potentiation (LTP) induced by repeated abnormal sensory signaling after peripheral nerve injury is a primary feature of chronic pain in the central nervous system, and the reinforcement of synaptic transmission is a core cellular mechanism of chronic pain [9]. Such long-term synaptic plasticity or central sensitization in the spinal cord and cortical areas is known to contribute to chronic pain [10]. In addition, changes in synaptic plasticity in the ACC are believed to be essential for the development and maintenance of chronic pain [9]. For instance, a brain imaging study found increased ACC neuronal activity in persistent pain conditions [11], and animal behavior studies employing acute and chronic pain models showed that the development of LTP increases the number of pre- and post-synaptic AMPA and NMDA receptors in the ACC and that drugs that inhibit ACC activity exert analgesic effects [12]. Therefore, these studies suggest that the ACC plays a vital role in processing pain-related information and in behavioral responses to various stimuli including noxious stimuli, inflammation, or nerve injury in humans and animals. In addition, a study utilizing a peripheral amputation model as well as an electrophysiological study reveals that excitatory transmission in the ACC is strengthened after peripheral nerve injury [13, 14]. In a rodent model of neuropathic pain, the development of allodynia is associated with synaptic potentiation in the ACC 1–2 weeks after nerve injury [15]. This finding suggests that neuropathic pain is associated with mechanisms underlying the development of LTP in the ACC. In addition, nerve damage could induce long-term enhancement of synaptic transmission in the ACC, which would not only increase the release of neurotransmitters from presynaptic terminals but also increase the number of postsynaptic receptors. Preventing, eliminating, or reducing enhancement of excitatory transmission could be applied as a new therapeutic method for suppressing chronic pain. Previous studies show that neurons in the ACC respond to noxious stimuli [16] and that lesioning the ACC significantly reduces acute and neuropathic pain responses [17]. However, inhibition of pain transmission does not prevent chronic pain-related synaptic strengthening, and drugs that suppress synaptic potentiation do not affect acute or physiological pain [12]. Therefore, it is necessary to identify new therapeutic targets for controlling chronic pain by investigating the induction and formation mechanisms of pain-related LTP. LTP in chronic pain can be classified into early LTP maintained by existing proteins and late LTP (L-LTP) maintained through protein synthesis [18, 19]. In particular, L-LTP contributes to long-term changes in the cortical circuit due to synaptic protein synthesis induced by peripheral nerve injury. Thus, the regulation of protein synthesis may be an avenue for controlling LTP, and LTP maintenance and formation could be regulated by modulating mammalian target of rapamycin (mTOR), which is involved in synaptic protein synthesis.

mTOR is a serine-threonine protein kinase that plays a critical role in cell proliferation and differentiation in the nervous system [20]. Activation of mTOR can be controlled through various stimuli (e.g., neurotransmitters, growth factors, cytokines, oxygen, and amino acids) [21]. Activation of mTOR affects transcription of mRNA in a variety of physiological and pathologic pain states, including inflammatory and cancer pain [22–26], by phosphorylating downstream factors such as eukaryotic initiation factor 4E binding protein 1 (4E-BP1) and p70 ribosomal S6 protein kinase (p70S6K) and is reported to be involved in signal transduction through regulation of protein synthesis [27]. mTOR can also regulate cell growth, differentiation, and synaptic plasticity by activating synaptic protein synthesis [19]. Thus, as LTP is maintained through synaptic protein synthesis [20], the modulation of synaptic protein synthesis through the regulation of mTOR activation may play an important role in LTP changes. Rapamycin, an mTOR inhibitor, has been used as an anticancer and immunosuppressive agent in the clinical treatment and has relatively few side effects compared with other drugs [26, 28]. Recent studies suggest that rapamycin, which regulates mTOR and its downstream effectors, could diminish chronic inflammatory, neuropathic, and cancer pain. Studies also report that mTOR activity is altered in a wide range of pathological states, such as cancer, cardiovascular diseases, and neurodegenerative disorders [26, 29, 30]. Pain behaviors (e.g., mechanical hypersensitivity) are reduced when rapamycin is intrathecally injected into hyperalgesia or inflammatory pain animal models or locally administrated in neuropathic pain animal models [22–24]. The inhibition of mTOR by treatment with the CCI-779, as confirmed by the decreased expression of downstream signaling molecules in the spinal cord and dorsal roots in a spared nerve injury animal model, exerts a pain-relieving effect [31]. Previous studies report that mRNA translation and local protein synthesis are regulated by inhibiting mTOR activation in several pain models [32, 33]. In addition, our laboratory found that rapamycin reduces the expression of downstream signaling molecules and synaptic proteins in the IC [34]. These previous studies strongly suggest that mTOR plays an important role in the modulation of long-term neuronal plasticity by preventing the establishment of LTP. So far, the mTOR pathway has been extensively studied in relation to cancer and described at the spinal cord level in relation to inflammatory and neuropathic pain, but only a few studies have been performed at the brain level. Moreover, there were no studies that have reported pain relief by controlling mTOR in the ACC of neuropathic pain models until now. It is necessary to conduct further studies at the brain level because the mTOR signaling pathway plays an important role in the neuronal plasticity involved in pain processing, such as the development of chronic pain. Therefore, the purpose of the present study was to investigate the association between pain caused by nerve injury and the mTOR signaling pathway in the ACC and to test the pain-relieving effect of rapamycin administration.

## Materials and Methods

### Experimental Animals

Adult male Sprague-Dawley rats that weigh 250–280 g (Harlan, Koatec, Pyeongtaek, Korea) were used in the present study. All animals were maintained under a 12-h light/dark cycle with free access to food and water throughout the study and were allowed to acclimate to the colony room for 7 days after arrival. All experimental protocols were in compliance with the National Institutes of Health guidelines and approved by the Institutional Animal Care and Use Committee of Yonsei University Health System (permit no. 2017-0148).

### Cannula Implantation

Rats were anesthetized with sodium pentobarbital (50 mg/kg, i.p.) and placed in a stereotaxic frame. Stainless steel guide cannulae (28-gauge) were bilaterally implanted into the ACC (AP 1.5 mm from bregma, ML ± 0.6 mm from midline, DV 1.5 mm beneath the surface of the skull). The cannulae were attached to the bone with stainless steel screws and acrylic cement. Dummy cannulae were inserted into the guide cannulae to avert clogging. Rats were allowed to recover for 7 days after cannula implantation.

### Neuropathic Surgery

A model of neuropathic pain was induced by ligation and cutting of the sciatic nerve branches [35]. Under sodium pentobarbital anesthesia, three terminal branches of the left sciatic nerve were exposed by direct incision of the skin and a section of the biceps femoris muscle. The tibial and sural nerves were tightly ligated with 4-0 black silk and cut, and the common peroneal nerve was left intact (nerve-injured; NP group). Surgical procedures for the sham-operated group were identical to those for the NP group except that the nerves were not injured. The neuropathic surgery and cannula implantation were all performed on the same day.

### Mechanical Allodynia Test

Rats were individually placed in small cages with a metal mesh floor and habituated for 15 min before the test. Mechanical allodynia was measured by assessing thresholds for each hind paw withdrawal from stimulation by an electronic von Frey filament (UGO Basile, Varese, Italy). A single, un-bending filament was vertically applied to the hind paw, and licking or rapid withdrawal of the hind paw was considered a positive response. Responses were measured seven times in 2–3-min intervals. The mechanical allodynia test was performed before nerve injury and on postoperative days (PODs) 1, 4, 7, and 14 by a researcher blinded to experimental conditions.

### Rapamycin Microinjection into the ACC

Rapamycin (R-5000; LC Laboratories, Woburn, MA, USA) was prepared in 0.06% dimethyl sulfoxide diluted in saline. Microinjections were performed on POD 7. Rapamycin (0.5 μl of 150, 300, and 600 nM per side over 1 min) or an equivalent amount of vehicle solution was microinjected into the ACC bilaterally via the injection cannulae, which were lowered 1 mm deeper into the ACC than the guide cannulae. The microinjection apparatus consisted of Hamilton syringes (1 μl) connected to the injection cannulae with PE-10 tubing. After injection, the injection cannulae were maintained in place for at least 1 min to allow for drug absorption into the brain and to minimize reflux along the track of the cannulae. Additional mechanical allodynia tests for confirming the effect of drug administration were performed before and 0.5, 1, 2, 4, 8, 12, and 24 h after rapamycin or vehicle solution microinjection into the ACC by a researcher blinded to microinjection substance.

### Western Blot Analysis/Tissue Collection and Immunoblotting

Two hours after microinjection of rapamycin or vehicle solution on POD 7, rats were anesthetized with isoflurane and decapitated for ACC tissue sample collection. The ACC (AP + 2.2 to − 1.2 from bregma) was dissected on ice using a rat brain matrix (RWD Life Science, Shenzhen, China). Three coronal brain slices (1 mm thick) containing the ACC were dissected using a surgical blade and rapidly frozen in liquid nitrogen. For protein extraction, samples were homogenized in lysis buffer (ProPrep; Intron Biotechnology, Pyeongtaek, Korea) containing phosphatase inhibitors (Phosstop; Roche, Mannheim, Germany) and incubated on ice for 2 h. Samples were then centrifuged at 15,000 rpm for 15 min at 4 °C, and supernatants were collected. Total protein concentrations were assessed with a spectrophotometer (Nano Drop ND-1000; NanoDrop Technologies Inc., Wilmington, DE, USA), and equal amounts of protein (30 mg per well) were denatured and loaded on 10% SDS-PAGE (for mTOR, p-mTOR, p70S6K, and p-p70S6K) and 15% SDS-PAGE (for 4E-BP1 and p-4E-BP1) (Bio-Rad, Hercules, CA, USA). Proteins were transferred onto a polyvinylidene difluoride membrane (Merck Millipore, Darmstadt, Germany). Phospho-proteins were immunodetected first followed by the corresponding total protein after the blot was stripped. For enhanced visualization and analysis of 4E-BP1 and p-4E-BP1, transferred proteins were fixed to the membrane by 0.05% glutaraldehyde in TBS-0.05% Tween-20 (TBST) for 15 min at room temperature [36]. Membranes were stained with Ponceau S (Sigma-Aldrich, St. Louis, MO, USA), and staining of the wells was visually compared. Membranes were blocked by incubation in 5% bovine serum albumin in TBST for 1 h at room temperature and incubation overnight at 4 °C with primary antibodies diluted in 5% bovine serum albumin in TBST. The following primary antibodies were used: mTOR (no. 2972, 1:1000; Cell Signaling Technology, Beverly, MA, USA), p-mTOR (Ser 2448, no. 2971, 1:500; Cell Signaling Technology), p70S6K (no. 2708, 1:1000; Cell Signaling Technology), p-p70S6K (Thr 389, no. 9205, 1:500; Cell Signaling Technology), 4E-BP1 (no. 9644, 1:1000; Cell Signaling Technology), p-4E-BP1 (Thr37/46, no. 2855, 1:500; Cell Signaling Technology), and β-actin (no. 3700, 1:15,000; Cell Signaling Technology). The next day, blots were washed three times in TBST and incubated with the appropriate anti-rabbit horseradish peroxidase-conjugated secondary antibody (no. 7074, 1:10,000; Cell Signaling Technology). Immunoreactive proteins were visualized by applying a chemiluminescent (ECL) detection reagent (GE Healthcare, Little Chalfont, UK) and observed using an LAS system (LAS 4000, GE Healthcare, Fuji Film Inc., Tokyo, Japan). Phospho-protein and total protein immunoreactivity was scanned and quantified using Multi-gage software (Fuji Film Inc., Tokyo, Japan). Each value was normalized to the respective β-actin band, and optical density was divided by that of the corresponding total protein band to yield a phospho-protein/total protein ratio.

### Immunohistochemistry

On POD 7, rats were deeply anesthetized with urethane (1.25 g/kg, i.p.) immediately after mechanical allodynia testing following microinjection of rapamycin or vehicle solution into the ACC and perfused transcardially with normal saline (0.9% NaCl) via the ascending aorta followed by a 4% solution of formaldehyde in 0.1 M sodium phosphate buffer (pH 7.4). Brains were removed, post-fixed in the same paraformaldehyde solution for 4 h at 4 °C, and immersed in a 10 to 30% (*w*/*v*) sucrose gradient in phosphate-buffered saline (PBS; pH 7.4) for 24–48 h at 4 °C for cryoprotection. After cryoprotection, brains were embedded in optimal cutting temperature compound (Sakura Finetek USA Inc., Torrance, CA, USA) and cut on a cryostat (Microm HM525; Thermo Scientific, Waltham, MA, USA) into 20-μm-thick coronal sections. Double immunofluorescence staining for PSD-95/AMPAR GluA1 subunits or PSD-95/NMDAR NR2B subunits was performed. Sections containing the ACC were blocked in PBS containing 0.3% Triton X-100 (PBST) and 5% normal donkey serum for 2 h at room temperature. For double immunofluorescence, sections were incubated overnight with a mixture of mouse anti-PSD-95 (1:500, ab2723, Abcam, Cambridge, UK) and rabbit AMPA GluA1 (1:500, ab31232, Abcam) or rabbit anti-NMDAR2B (1:500, ab65783, Abcam) antibodies in PBST containing 5% normal donkey serum at 4 °C. All sections were rinsed with PBST (5 min, three times) after each step. After rinsing, sections were incubated with Cy3-conjugated donkey anti-mouse IgG (H + L) (1:200, 715-165-150, Jackson ImmunoResearch, West Grove, PA, USA) or Alexa Fluor 488-conjugated donkey anti-rabbit IgG (H + L) (1:200, 711–545-152, Jackson ImmunoResearch) secondary antibody for 2 h at room temperature. Sections were mounted with Vectashield containing DAPI to label nuclei (Vector Laboratories, Youngstown, OH, USA). In each experiment, sections from different groups were stained at the same time.

High-magnification fluorescent images were taken with a Zeiss LSM 700 confocal microscope (Carl Zeiss, Jena, Germany). Quantitative assessment of PSD-95 and AMPAR or NMDAR immunoreactivity was performed using the ImageJ 1.46r software program (National Institutes of Health, Bethesda, Maryland, USA). In addition, the total number of pixels of the stained area was quantified and expressed as immunodensity. These measurements were performed using two sections randomly selected from the five serial sections of the ACC from each animal. Immunodensities were averaged to obtain a single value for each rat.

### Optical Imaging

Before imaging, rats were deeply anesthetized with urethane (1.25 g/kg, i.p.) on POD 7 and given atropine (5 mg/kg, i.p.) to suppress mucus secretion and dexamethasone sulfate (1 mg/kg, i.p.) to reduce swelling of the brain. Rats were then placed in a custom-made stereotaxic frame to stabilize the head during optical imaging. Lidocaine was applied to the skin, and a craniotomy was made that extended AP + 6 to − 2 mm from bregma and ML ± 4 mm from midline. The dura over the ACC was carefully removed. To expose the ACC, double closure of the superior sagittal sinus was performed using 4–0 black silk, and the superior sagittal sinus was then cut with microscissors and separated from the cortex. If slight bleeding occurred, it was easily controlled using a saline-moistened cotton compress. This surgical procedure was slightly modified from that described for a rat pinealectomy study and an electrophysiological study [37, 38]. The exposed ACC was stained using voltage-sensitive dye (di-2-ANEPEQ, 50 mg/mL in saline; Molecular Probes, Eugene, OR, USA) for 1 h and carefully rinsed with saline. Imaging was performed before and after application of 600 nM rapamycin or vehicle solution directly to the exposed cortex for 30 min. Heart rate was monitored by electrocardiography, and body temperature was maintained at 36 °C with a rectal probe and heating pad system (Homeothermic Blanket Control Unit; Harvard Apparatus, Holliston, MA, USA).

A pair of stainless steel electrodes was inserted into the left hind paws where the electronic von Frey filaments had been applied during behavioral testing. Stimulation of the hind paw was done with square pulses (width 0.1 ms, interstimulus interval 5 s, intensity 2.5 mA) using a stimulus isolation unit (World Precision Instruments, Sarasota, FL, USA). Changes in fluorescence after peripheral stimulation were measured using a high-resolution CCD camera (Brainvision Inc., Tokyo, Japan) equipped with a dichroic mirror with a 510–550-nm excitation filter and 590-nm absorption filter. A tungsten halogen lamp (150 W) was used for excitation of fluorescence. The imaging area was 6.4 × 4.8 mm^2^ and consisted of 184 × 124 pixels. The fluorescence intensity during each trial was detected for approximately 940 ms under an optical microscope (Leica Microsystems Ltd., Heerbrugg, Switzerland) equipped with a ×1 objective and ×1 projection lens. Optical signals were acquired at a rate of 3.7 ms/frame, and averages of 20 trials were recorded by an optical imaging recording system (MiCAM02; Brainvision Inc.). Optical imaging acquisition was triggered by electrocardiogram signals using a stimulus/non-stimulus subtraction method. After optical imaging, rats were euthanized by an overdose of urethane. To normalize the value of each pixel, the ratio of the intensity of fluorescence (ΔF) in each pixel relative to the initial fluorescence intensity (F) was expressed as a fractional change (ΔF/F). Amplitudes and excitatory areas of optical signals were measured using a spatial filter (9 × 9 pixels) to reduce artifacts caused by vibration and brain movements. Using captured images, fractional changes in optical signals (i.e., optical intensity) and areas of activation were quantified. Changes in optical intensity in the ACC were expressed as a percentage of fractional change in fluorescence (%Δ*F*/*F*). Activated areas were analyzed using the activated pixel number divided by the total pixel number in the region of interest (AP + 2.2 to + 0.2 mm from bregma) × 100. Data were collected and analyzed with BV Analyzer software (Brainvision Inc.).

### Statistical Analysis

Statistical analyses were performed using SPSS 20.0 software (IBM Corporation, Armonk, NY, USA). Behavioral data were analyzed by two-way repeated measures analysis of variance (ANOVA) followed by Bonferroni’s test for post hoc comparisons. Western blotting and immunohistochemistry data were analyzed by one-way ANOVA followed by Tukey’s test for multiple comparisons. Optical imaging data were analyzed by paired *t* test. All values are expressed as mean ± standard error of the mean (SEM). Values of *p* < 0.05 were considered statistically significant.

## Results

### Development of Mechanical Allodynia Induced by Nerve Injury

**Fig. 1:**
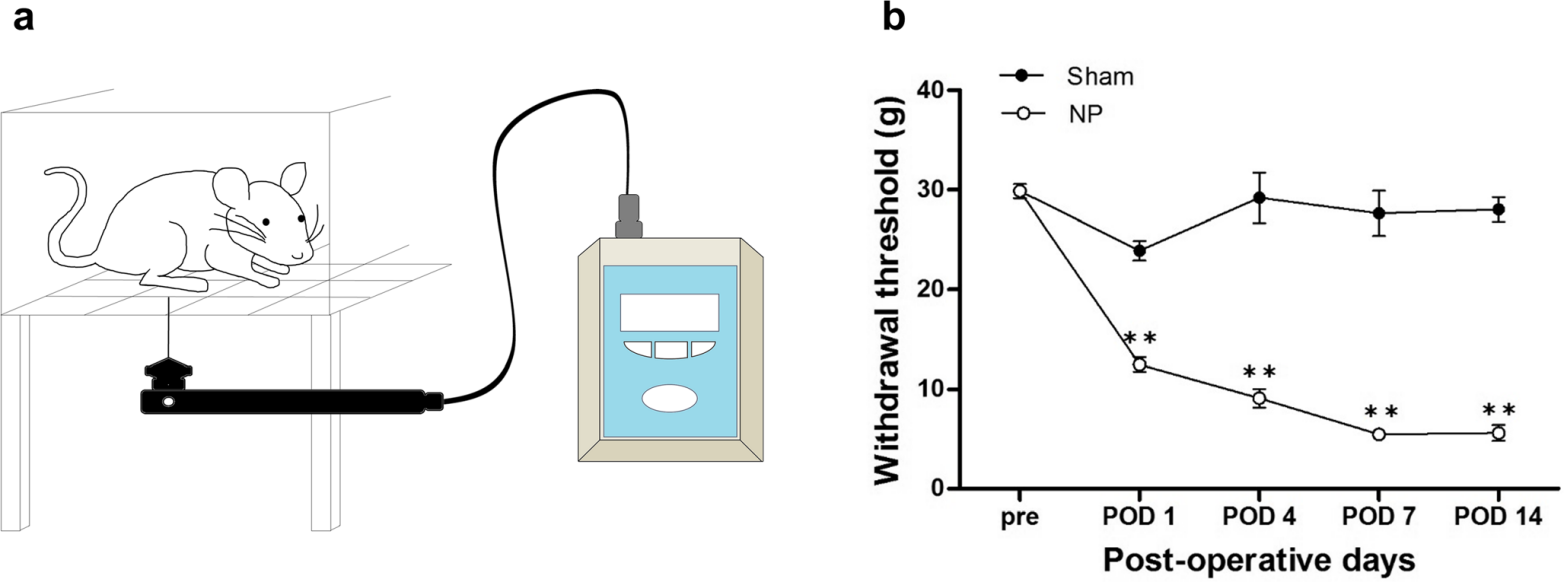
Electronic von Frey test and development of mechanical allodynia. **a** Illustration of electronic von Frey test for assessing mechanical withdrawal threshold. Rats were individually placed on a metal mesh floor in small plastic cages. A filament was vertically applied to a hind paw, and force was measured until paw withdrawal occurred and was displayed by the device. **b** Development of mechanical allodynia in nerve-injured (NP) and sham-injured (Sham) rats. After nerve injury, significantly lower mechanical withdrawal thresholds were observed in the NP group on POD 1, 4, 7, and 14 compared with those in the Sham group. Data are presented as mean ± standard error of mean (SEM). **p < 0.01 vs. Sham, two-way repeated measures analysis of variance (ANOVA) followed by Bonferroni test

To confirm the development of mechanical allodynia, we examined hind-paw withdrawal threshold in our neuropathic pain animal model using the electronic von Frey 1, 4, 7, and 14 days after neuropathic pain surgery (Fig. 1a). From POD 1 to POD 14, withdrawal threshold was significantly decreased in the NP group (*n* = 6) compared with the control (Sham) group (*n* = 6; for nerve injury *F*_1,70_ = 703.223, *p* < 0.001; for POD *F*_4,70_ = 55.943, *p* < 0.001; for nerve injury × POD *F*_4,70_ = 54.038, *p* < 0.001; two-way repeated measures ANOVA followed by Bonferroni test, Fig. 1b). However, there was no significant difference between groups before surgery.

### Nerve Injury Activates mTOR Signaling in the ACC

**Fig. 2:**
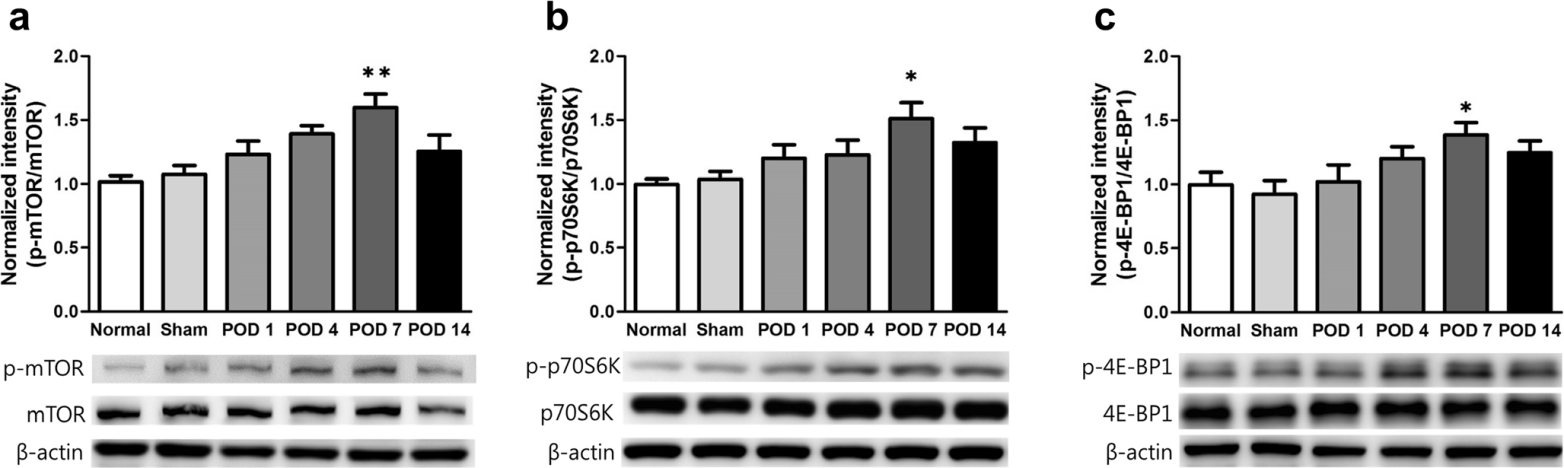
Activation of mTOR and its downstream effectors in the ACC after nerve injury. **a**–**c** Expression levels of p-mTOR, p-p70S6K, and p-4E-BP1 after nerve injury. Levels of p-mTOR, p-p70S6K, and p-4E-BP1 gradually increased after nerve injury as shown by western blot analysis. Seven days after nerve injury (POD 7), p-mTOR, p-p70S6K, and p-4E-BP1 expression were significantly up-regulated compared with the normal group, but all phospho-protein levels declined 14 days after nerve injury (POD 14) as compared with the POD 7 group. Total protein levels in all groups did not change over time. Data are presented as mean ± SEM. *p < 0.05, **p < 0.01 vs. normal, one-way ANOVA followed by Tukey’s multiple comparison test

Based on our behavioral results, we hypothesized that mTOR signaling in the ACC would be altered during the development of chronic pain after nerve injury. To confirm this hypothesis, we examined the mTOR signaling pathway using western blot analysis. We measured total and phosphorylated protein levels of mTOR and its downstream effectors, P70S6K and 4E-BP1, in the ACC 1, 4, 7, and 14 days after nerve injury (POD 1, POD 4, POD 7, and POD 14) as well as in normal and sham injury groups. We found that phosphorylated mTOR (p-mTOR) was significantly up-regulated in the POD 7 group compared with the normal group (*n* = 6; *F*_5,30_ = 5.456, *p* = 0.001, one-way ANOVA followed by Tukey’s multiple comparison test, Fig. 2a). Furthermore, levels of p-p70S6K and p-4E-BP1 were significantly elevated in POD 7 group compared with the normal group (*n* = 6; *F*_5,30_ = 3.669, *p* = 0.010 and *F*_5,30_ = 2.908, *p* = 0.029, respectively, one-way ANOVA followed by Tukey’s multiple comparison test, Fig. 2b, c), whereas there were no group differences in the total forms of these proteins (*p* = 0.849, one-way ANOVA). Thus, activation of mTOR and its downstream effectors in the ACC gradually increased after nerve injury and peaked on POD 7. However, expression of p-mTOR, p-p70S6K, and p-4E-BP1 declined slightly between POD 7 and POD 14.

### Microinjection of Rapamycin into the ACC Diminishes Mechanical Allodynia After Nerve Injury

**Fig. 3:**
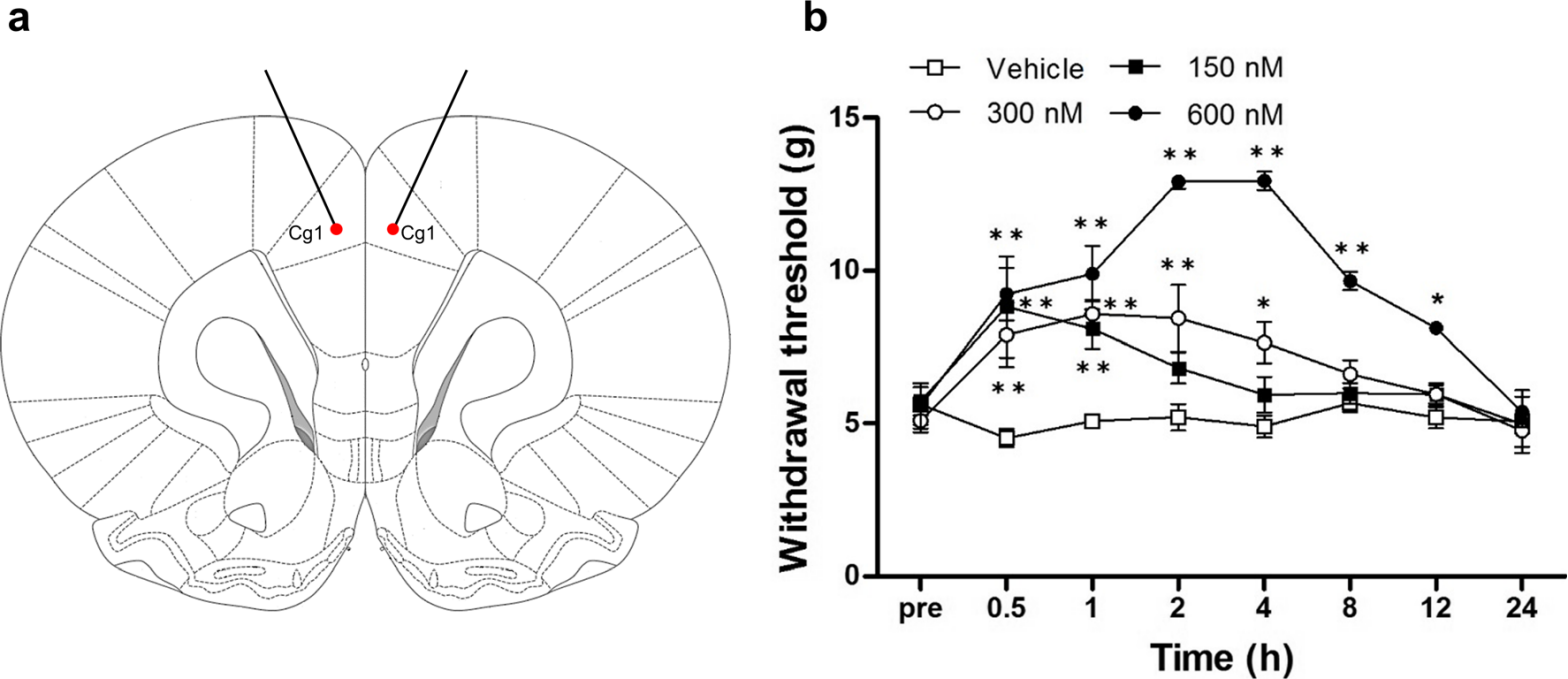
Microinjection of rapamycin into the ACC attenuates mechanical hypersensitivity. **a** Stereotaxic location of rapamycin microinjection into the ACC. Red dots point at microinjection sites and Cg1 indicates cingulate cortex, area 1 in the rat brain atlas. Stainless steel guide cannulae for drug microinjection were bilaterally implanted into the ACC. **b** Changes in paw withdrawal threshold to mechanical stimulation after microinjection of rapamycin or vehicle on POD 7. Significant differences in withdrawal threshold between 150 nM rapamycin and vehicle groups were observed between 0.5 and 1 h after microinjection. Withdrawal thresholds in the 300 nM rapamycin group were significantly elevated between 0.5 and 4 h after microinjection. The most pronounced changes in withdrawal threshold were observed in the 600 nM rapamycin group, with significant changes observed between 0.5 and 8 h after microinjection. Data are presented as mean ± SEM. *p < 0.05; **p < 0.01 vs. vehicle, two-way repeated measures ANOVA followed by Bonferroni test

After the establishment of mechanical allodynia, rapamycin (150, 300, or 600 nM; *n* = 8) or vehicle (*n* = 8) was injected into the ACC (Fig. 3a). Mechanical sensitivity was examined 0.5, 1, 2, 4, 8, 12, and 24 h after injection. All rapamycin-injected groups showed a reduced response to mechanical stimulation as measured in the von Frey test (*n* = 8; for drug (vehicle or rapamycin): *F*_3,224_ = 74.272, *p* < 0.001; for time (after drug injection): *F*_7,224_ = 22.196, *p* < 0.001; for drug × time: *F*_21,224_ = 16.427, *p* < 0.001; two-way repeated measures ANOVA followed by Bonferroni test, Fig. 3b). The response to mechanical stimulation in the 150 nM rapamycin group significantly increased from 0.5 to 1 h (0.5 and 1 h: *p* < 0.001 vs. vehicle group, two-way repeated measures ANOVA followed by Bonferroni test), and the analgesic effect of 300 nM rapamycin persisted from 0.5 to 4 h (0.5 to 2 h: *p* < 0.001, 4 h: *p* = 0.026 vs. vehicle group, two-way repeated measures ANOVA followed by Bonferroni test). However, 600 nM rapamycin was the most effective dose (0.5 to 8 h: *p* < 0.01, 12 h: *p* = 0.013 vs. vehicle group, two-way repeated measures ANOVA followed by Bonferroni test). No change in withdrawal threshold to mechanical stimuli was observed in the vehicle group during the 24 h after microinjection (*p* = 0.749, two-way repeated measures ANOVA).

### Microinjection of Rapamycin into the ACC Blocks Activation of mTOR and its Downstream Effectors in the ACC After Nerve Injury

**Fig. 4:**
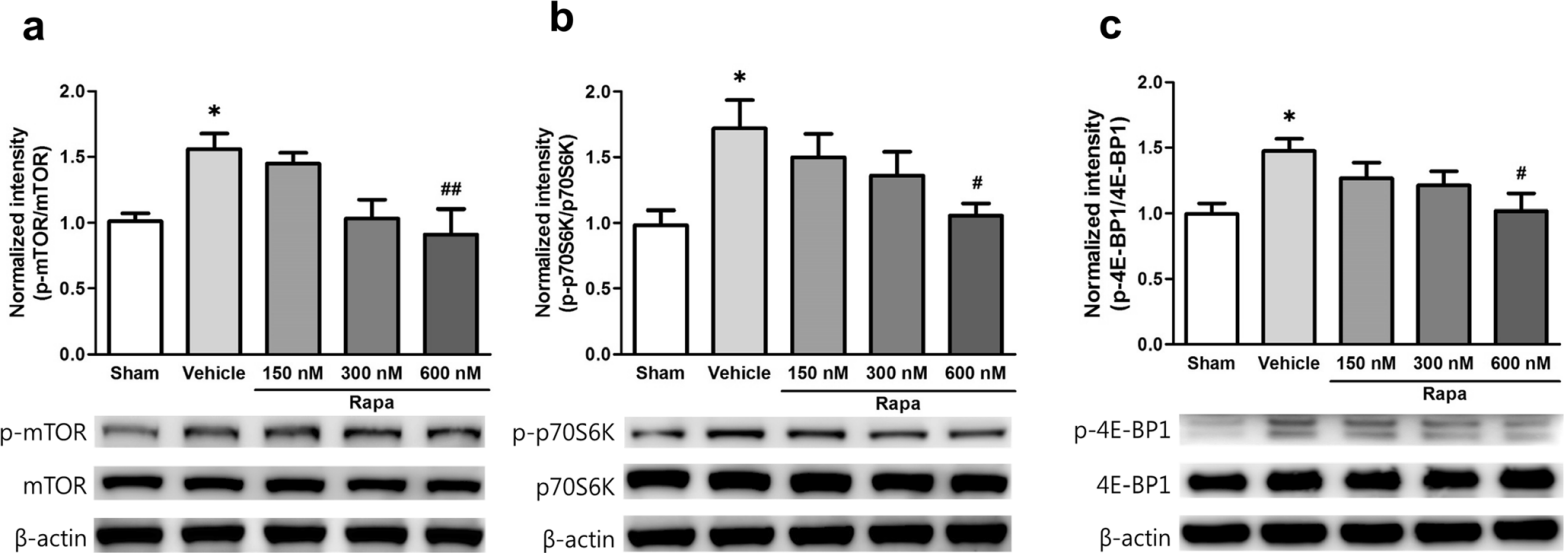
Microinjection of rapamycin into the ACC suppresses activation of mTOR and its downstream effectors after nerve injury. **a**–**c** Expression of p-mTOR, p-p70S6K, and p-4E-BP1 after microinjection of rapamycin. Levels of p-mTOR, p-p70S6K, and p-4E-BP1 were significantly increased in the vehicle group compared with the Sham group and significantly decreased in all rapamycin groups (Rapa) compared with the vehicle group in a dose-dependent manner. Levels of p-mTOR, p-p70S6K, and p-4E-BP1 were most prominently reduced in the 600 nM rapamycin group compared with the vehicle group. Total protein levels were similar among groups. Data are presented as mean ± SEM. *p < 0.05; **p < 0.01 vs. Sham; ^#^*p* < 0.05 vs. vehicle, one-way ANOVA followed by Tukey’s multiple comparison test

We next confirmed changes in the mTOR signaling pathway after rapamycin injection by western blot analysis. As the highest paw withdrawal threshold was reached 2 h after 600 nM rapamycin injection and persisted to 4 h, we collected ACC tissue 2 h after rapamycin or vehicle microinjection on POD 7 and measured the expression of mTOR and its downstream effectors. The levels of p-mTOR, p-p70S6K, and p-4E-BP1 in the vehicle group were significantly higher than those in the Sham group (p-mTOR *F*_4,35_ = 5.164, *p* = 0.035; p-p70S6K *F*_4,35_ = 3.568, *p* = 0.022; and p-4E-BP1 *F*_4,35_ = 3.313, *p* = 0.027, one-way ANOVA followed by Tukey’s multiple comparison test, Fig. 4a–c), and the rapamycin groups tended to exhibit a gradual decrease in phospho-protein levels in a dose-dependent manner. In particular, the level of p-mTOR in the 600 nM rapamycin group was significantly lower than that in the vehicle group (*p* = 0.008, one-way ANOVA followed by Tukey’s multiple comparison test, Fig. 4a). In addition, p-p70S6K and p-4E-BP1 expression was significantly lower than that in the vehicle group (*p* = 0.048 and *p* = 0.038, respectively, one-way ANOVA followed by Tukey’s multiple comparison test, Fig. 4b, c), but there were no changes in total protein levels (*p* = 0.632, one-way ANOVA).

### Inhibition of mTOR Suppresses Synaptic Protein Expression in the ACC After Nerve Injury

**Fig. 5:**
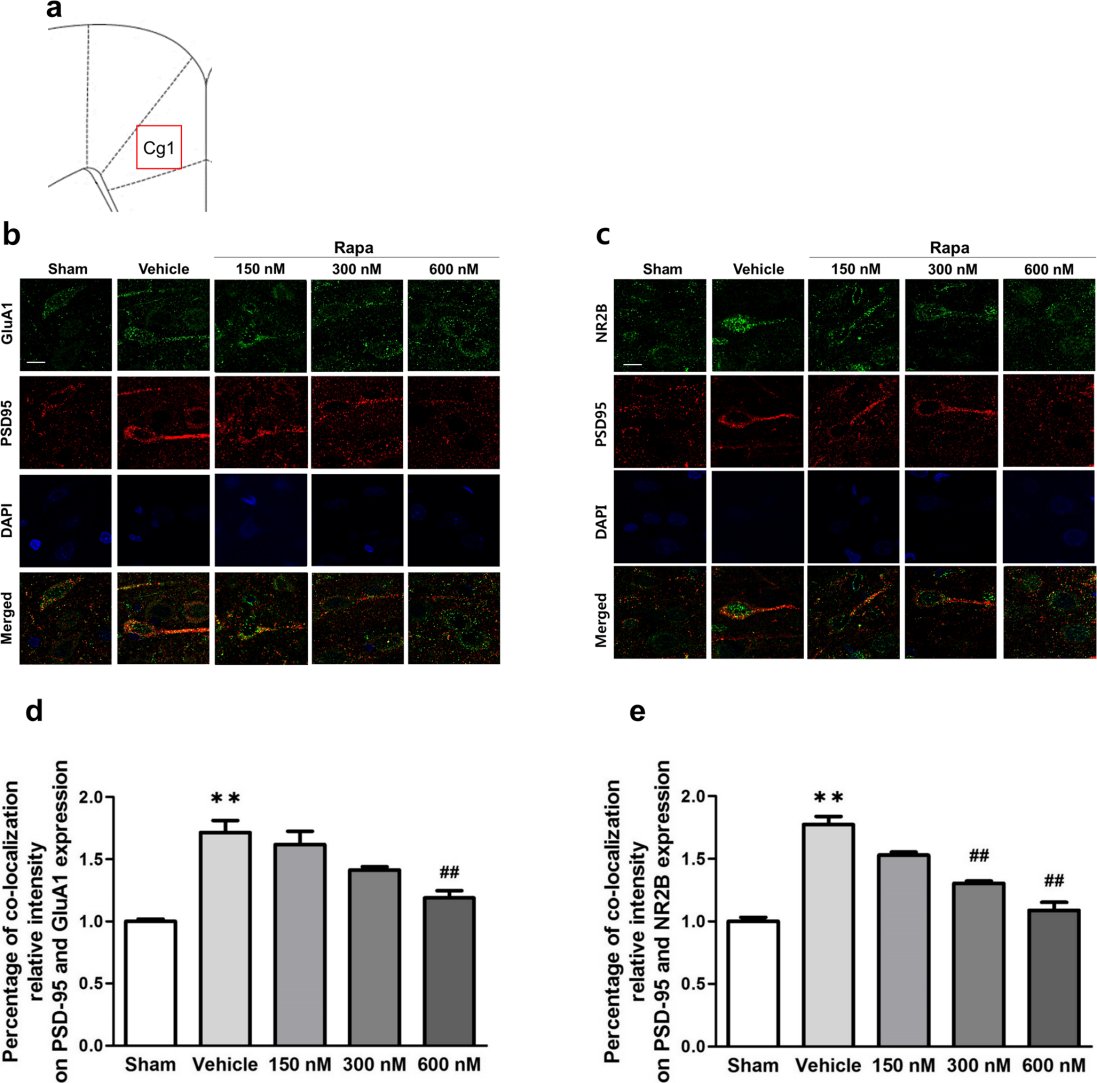
Synaptic protein expression after inhibition of mTOR signaling in the ACC. **a** Location of the ACC used for staining analysis. Cg1 indicates cingulate cortex, area 1 in the rat brain atlas and red squire indicates location for staining analysis. **b** Different sets of ACC tissue treated with rapamycin (Rapa) or vehicle on POD 7 were subjected to immunofluorescence staining with anti-PSD-95 (red), anti-GluA1 (green) or anti-NR2B (green), and DAPI (blue). Representative immunofluorescence staining of PSD-95, AMPA receptor GluA1 subunit, and nuclei (DAPI). **c** Representative immunofluorescence staining of PSD-95, NMDA receptor NR2B subunit, and nuclei (DAPI). Scale bar = 10 μm. **d** Percentage of co-localization relative intensity of PSD-95 and AMPA GluA1 receptor subunit expression. The percentage was significantly higher in the vehicle group than in the Sham group, and the percentages in the Rapa groups declined in a dose-dependent manner. The 600-nM group showed a significant decrease compared with the vehicle group. **e** Percentage of co-localization relative intensity of PSD-95 and NMDA NR2B receptor subunit expression. The percentage was significantly higher in the vehicle group than in the Sham group, and the percentages in the Rapa groups declined in a dose-dependent manner. The 300- and 600-nM groups showed significant decreases compared with the vehicle group. Data are presented as mean ± SEM. **p < 0.01 vs. Sham; ^**##**^*p* < 0.01 vs. vehicle, one-way ANOVA followed by Tukey’s multiple comparison test

To determine the effect of rapamycin-induced inhibition of mTOR signaling on synaptic protein synthesis, we performed immunofluorescence staining. We observed changes in the co-localization relative intensity of PSD-95 and glutamate receptors (AMPAR GluA1 subunit or NMDAR NR2B subunit) in the ACC 2 h after rapamycin or vehicle microinjection on POD 7. Figure 5a shows location of the ACC used for staining analysis. Cg1 indicates cingulate cortex, area 1 in the rat brain atlas. AMPAR-PSD-95 and NMDAR-PSD-95 co-localization were markedly increased in ACC layer II/III after peripheral nerve injury (Fig. 5b, c). Moreover, rapamycin injection resulted in a dose-dependent reduction in the percentage of co-localization relative intensity of PSD-95 and glutamate receptors in the ACC compared with that in the vehicle group. Specifically, AMPAR-PSD-95 co-localization was significantly lower in the 600 nM group than in the vehicle group (*F*_4,25_ = 29.331, *p* < 0.001, one-way ANOVA followed by Tukey’s multiple comparison test, Fig. 5d), and NMDAR-PSD-95 co-localization was significantly lower in the 300 nM and 600 nM groups than in the vehicle group (*F*_4,25_ = 46.517, *p* < 0.001, one-way ANOVA followed by Tukey’s multiple comparison test, Fig. 5e).

### Inhibition of mTOR Reduces Excitability in the ACC Induced by Peripheral Stimulation

**Fig. 6:**
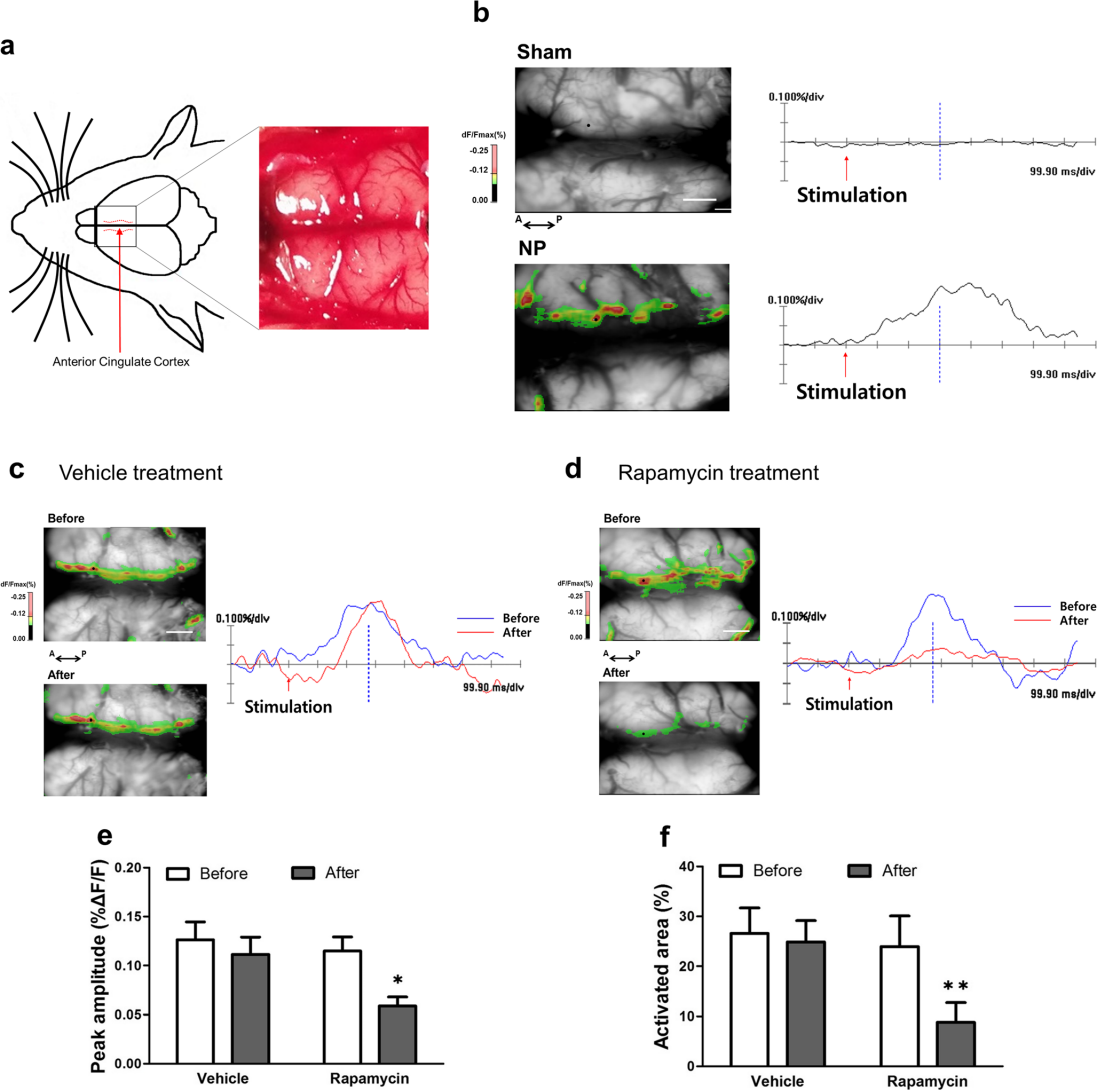
Changes in optical signals in the ACC. **a** Dorsal view of the exposed cortex and representative image. The inner area of the dotted red lines indicates the ACC region. **b** Representative optical images and signals in the ACC from sham-injured (Sham, upper) and nerve-injured (NP, lower) rats. Red arrows indicate time point of electrical stimulation. NP rats showed a larger activated area in the ACC and increased peak amplitude after peripheral stimulation (2.5 mA) compared with Sham rats. Scale bar = 1 mm. **c**, **d** Optical signals from NP rats before and after vehicle or 600 nM rapamycin treatment. Blue lines mean before treatment and red lines mean after treatment in the optical signals. **e**, **f** Peak amplitudes and activated areas before and after vehicle or rapamycin treatment following peripheral electrical stimulation (2.5 mA). The peak amplitude and activated area induced by peripheral electrical stimulation after rapamycin treatment were significantly lower than those before rapamycin treatment. However, they were similar before and after vehicle treatment. Data are presented as mean ± SEM. *p < 0.05; **p < 0.01, paired *t* test

Optical imaging using voltage-sensitive dye is a convenient approach to measuring cortical activity after peripheral stimulation. In the present study, we imaged the ACC of nerve-injured rats to detect optical signal changes after electrical stimulation of the hind paw. To investigate differences in spatiotemporal patterns in the ACC of sham (Sham) versus nerve-injured (NP) rats, we exposed the cortex (Fig. 6a) and obtained representative optical images after electrical stimulation of the contralateral hind paw (Fig. 6b). Color-coded images in the left side of Fig. 6b are optical signals of the activated ACC induced by peripheral stimulation based on fractional changes in reflected light intensity (%Δ*F*/*F*), and wave forms in the right side of Fig. 6b show optical responses at specific points in the ACC. Whereas the ACC of NP rats showed optical responses, little change in optical signals and wave forms in the ACC were found after 2.5 mA electrical peripheral stimulation in Sham rats.

Optical images of responses to peripheral stimulation before and after rapamycin treatment are shown in Fig. 6c, d. Figure 6c, d shows representative images before and after vehicle treatment or 600-nM rapamycin treatment, respectively, following 2.5-mA electrical peripheral stimulation in nerve-injured rats. As we found a maximal pain-relieving effect of 600 nM rapamycin injection, we used 600 nM rapamycin to validate neural activity in the ACC. After rapamycin treatment, neuronal activity in the ACC was strikingly reduced compared with that before treatment. Peak amplitudes induced by peripheral electrical stimulation were reduced after rapamycin treatment compared with before rapamycin treatment (*t*_5_ = 5.07, *p* = 0.003, paired *t* test, Fig. 6e). Also, the area of activation was reduced after rapamycin treatment compared with before rapamycin treatment (*t*_5_ = 8.069, *p* < 0.001, paired *t* test, Fig. 6f). By contrast, peak amplitude and activated area were not significantly different before and after vehicle treatment (*p* = 0.838 and *p* = 0.443 respectively, paired *t* test).

## Discussion

Changes in synaptic plasticity in many areas of the central nervous system are involved in pain processing and chronic pain, including the spinal cord, cortical areas, and subcortical areas [10, 12]. Previous studies show that changes of neural plasticity in the ACC are associated with persistent pain and fear memory in a mouse model [39]. ACC neurons respond to nociceptive and non-nociceptive mechanical or thermal somatosensory stimuli as well as emotional aspects of pain [16, 40, 41]. In particular, nociceptive information is projected to the ACC from other pain-related regions, such as the medial thalamus, amygdala, and other pain-related area of the cortex [42].

Recently, mTOR has been found to control cancer, inflammatory, and neuropathic pain [24, 26, 31, 43]. These studies provide evidence that mTOR plays an important role in pain management but have mainly focused on mechanisms at the spinal level. The present study validated the role of mTOR signaling at the brain level by examining mechanical hyperalgesia and changes in the expression of activated mTOR (p-mTOR) and its downstream effectors (p-p70S6K and p-4E-BP1) in the ACC after nerve injury in an animal model of neuropathic pain. Our results suggest that the development of neuropathic pain induced by peripheral nerve injury is associated with increased activity of mTOR and its downstream effectors in the ACC. Previous studies report increased activity of mTOR and its downstream effectors in the spinal cord dorsal horn in the chronic constriction injury model [44], the rostral ventromedial medulla in the spared nerve injury model [45], and the IC in the nerve-injured model [34]. Also, another study reports increased expression of p-mTOR and p-p70S6K in the ACC of a formalin-induced inflammatory animal model [46]. Together with previous studies, our results demonstrate that the mTOR-dependent signaling pathway in the ACC is involved in the development of chronic pain. In addition, the expressions of activated mTOR and its downstream effectors on POD 14 were less than on POD 7. The differences in the expression levels on POD 14 and POD 7 might indicate that the mTOR signaling pathway in the ACC becomes less effective in maintaining nerve injury-induced chronic pain.

Previous electrophysiological studies show that rapamycin, an mTOR inhibitor, inhibits L-LTP in the hippocampus when delivered during LTP induction but not after LTP formation [18, 47]. Also, intrathecal injection of rapamycin reduces mechanical hyperalgesia behaviors and decreases phosphorylation of mTOR and its downstream effectors in the spinal cord dorsal horn in chronic constriction injury and spinal cord injury animal models [22, 44]. These results indicate that neuropathic pain could be regulated by rapamycin. In the present study, mechanical allodynia was significantly reduced after microinjection of rapamycin into the ACC, and levels of phosphorylation of mTOR, p70S6K, and 4E-BP1 were significantly decreased in a dose-dependent manner. In particular, 600 nM rapamycin was the most effective dosage for reducing mechanical allodynia and phosphorylation of mTOR, p70S6K, and 4E-BP1. Previous studies have reported that rapamycin decreases pain behaviors in a dose-dependent manner without a change in locomotor activity and apparent adverse effects [48–50]. Thus, these results suggest that rapamycin reduces pain-related behaviors by inhibiting the activation of mTOR.

mTOR regulates synaptic protein transcription via many factors [51]. Activation of mTOR regulates the phosphorylation of downstream effectors such as p70S6K and 4E-BP1, which in turn regulate mRNA translation and protein synthesis [27, 52] and thereby affect protein translation [22]. Local protein synthesis in axons and dendrites contributes to regulating long-lasting synaptic plasticity, neurite development, and response to nerve injury [53, 54]. It is well-known that mTOR regulates local transcription of mRNA through phosphorylation of p70S6K and 4E-BP1 [47]. Phosphorylation of p70S6K mediates translation initiation and polypeptide extension, whereas phosphorylation of 4E-BP1 reduces cap-dependent transcription by inhibiting eukaryotic initiation factor 4E (eIF4E) [52]. Activation of mTOR signaling induces dendritic protein synthesis, including the synthesis of postsynaptic glutamate receptors and postsynaptic density proteins necessary for maintaining long-lasting forms of synaptic plasticity, especially L-LTP [52, 55]. A previous study has shown that intrathecally administered rapamycin reduces expression levels of activated mTOR and its downstream effectors in the spinal dorsal horn at 7 days, but not 14 days, after nerve injury [44]. This finding agrees well with our observations showing that expression levels of p-mTOR in the ACC increased on 7 days after nerve injury and declined thereafter. We have also observed that inhibition of mTOR signaling by rapamycin injected bilaterally into the ACC on POD 7 reduced nerve injury-induced pain behavior. Thus, it is suggested that during the development of chronic pain, activation of mTOR signaling is necessary to express pain behaviors. In addition, activation of postsynaptic receptors affects the development of chronic hyperalgesia to noxious stimuli [56]. Post-synaptic density (PSD), which binds to postsynaptic receptors and adhesion molecules, is maintained by an abundant set of scaffold proteins and has a role in mediating activation of synaptic receptors for biochemical signaling events in post-synaptic neurons [57]. In particular, the interaction between the NMDA receptor NR2B subunit and PSD-95 plays an important role in synaptic plasticity in the central nervous system underlying the development of neuropathic pain after peripheral nerve injury [14]. The AMPA receptor GluA1 subunit also contributes to pain-related LTP expression in the ACC [58]. Thus, it is possible that the mTOR pathway induces long-term changes in synaptic efficacy by facilitating interaction of glutamate receptors with PSD proteins, leading to the long-lasting synaptic plasticity associated with chronic pain.

Here, we provide strong evidence that mTOR contributes to neuropathic pain induced by peripheral nerve injury by increasing the excitability of pyramidal neuron in the ACC. Our immunofluorescence staining showed that the expression of PSD-95 was increased at apical dendrites of ACC pyramidal neurons after nerve injury and confirmed increased interactions between PSD-95 and glutamate receptors (AMPAR GluA1 and NMDAR NR2B subunits). In addition, rapamycin administration into the ACC decreased the expression of and interaction between these synaptic proteins. These results demonstrate that rapamycin suppresses the activity of mTOR and its downstream effectors, thereby decreasing translation and synthesis of mTOR-mediated synaptic proteins. Because both the ACC and primary somatosensory cortex, which are located in the pain ascending pathway, receive nociceptive inputs from the medial thalamus, the increased activity of pyramidal cells in both areas reflects their participation in a cerebral cortical circuit involved in chronic pain. In fact, electrophysiological studies show the activation of many pyramidal neurons of ACC layer II/III after nerve injury [59, 60], and some studies show that inhibiting the hyperactivity or plasticity of pyramidal neurons in the ACC lessens the development of chronic pain [59, 61]. Combining our current results with those of previous studies, we speculated that rapamycin modulates the interaction between GluA1-PSD-95 and NR2B-PSD-95 by regulating the mTOR signaling pathway that controls synaptic plasticity in the ACC. In other words, mTOR signaling in the ACC is an important component in neuropathic pain induced by nerve damage.

We performed optical imaging using voltage-sensitive dye as an additional means to verify neuronal activation of the ACC in neuropathic pain and to demonstrate changes in neuronal activity after rapamycin treatment. This electrophysiological technique allows the recording of membrane potential changes to provide visual confirmation of the activity of the neuronal population [62]. We delivered electrical stimuli to hind paws to observe changes in nerve activity after rapamycin treatment in the ACC of an animal nerve injury model. Our results indicate that the ACC of neuropathic rats was extensively activated in response to peripheral electrical stimulation, and this ACC activation was diminished by rapamycin. In other words, excitability in the ACC after nerve injury decreased after treatment with an mTOR inhibitor. Previous studies report similar results in the IC, which is also involved in pain processing [34]. These findings, together with those of previous studies, indicate that rapamycin could reduce the response to peripheral stimulation before the development of LTP. The purpose of this study was to validate the efficacy of inhibiting mTOR activation in the ACC in relieving neuropathic pain caused by nerve injury. Consistent with previous studies, our findings suggest that protein synthesis, which maintains a chronic pain state, is suppressed by inhibiting the activity of mTOR prior to LTP development. However, the effect of pain relief did not persist more than 24 h after rapamycin microinjection; therefore, future studies investigating the maintenance of this effect are needed. In conclusion, the present study suggests that the mTOR pathway in the ACC is an important avenue for controlling neuropathic pain produced by peripheral nerve damage and provides evidence for a vital role of synaptic plasticity in the ACC in the processing of pain.
